# Exploring the causal links between cigarette smoking, alcohol consumption, and aneurysmal subarachnoid hemorrhage: a two-sample Mendelian randomization analysis

**DOI:** 10.3389/fnut.2024.1397776

**Published:** 2024-09-13

**Authors:** Heng Lin, Yanqing Yin, Jie Li, Siwei Liu, Xiaoao Long, Zhuangbin Liao

**Affiliations:** Department of Cerebrovascular Surgery, Affiliated Hospital of Guangdong Medical University, Zhanjiang, China

**Keywords:** cigarette smoking, alcohol consumption, aneurysmal subarachnoid hemorrhage, Mendelian randomization investigation, lifestyle behaviors

## Abstract

**Background:**

Aneurysmal subarachnoid hemorrhage (aSAH) represents a critical health concern characterized by elevated mortality and morbidity rates. Although both genetic predisposition and lifestyle choices influence aSAH susceptibility, understanding the causative associations between cigarette smoking, alcohol consumption, and aSAH risk remains imperative. Mendelian randomization (MR) offers a robust methodological framework for dissecting these associations, leveraging genetic variants as instrumental variables.

**Objective:**

In this study, a two-sample Mendelian randomization (TSMR) approach was employed to elucidate the causal connections between genetically determined cigarette smoking, alcohol consumption, and aSAH risk.

**Methods:**

Genetic instruments associated with cigarette smoking and alcohol consumption were sourced from the genome-wide association study (GWAS) and Sequencing Consortium of Alcohol and Nicotine use (GSCAN). Using a genome-wide association study (GWAS) dataset that encompassed aSAH cases and controls of European ancestry, TSMR, which utilized the inverse variance weighting (IVW) method, was employed to estimate the causal effects. Rigorous criteria were applied for selecting instrumental variables to ensure a robust Mendelian randomization analysis.

**Results:**

A significant causal association was found between genetically determined cigarette smoking and an increased risk of aSAH, with a 1-standard deviation (SD) increase in cigarette use genetically linked to a 96% relative risk elevation [OR-IVW = 1.96, 95% confidence interval (CI) = 1.28–3.01, *p* = 0.0021]. However, genetically determined alcohol consumption did not exhibit a statistically significant association with aSAH risk (OR-IVW = 1.22, 95% CI = 0.61–2.45, *p* = 0.578).

**Conclusion:**

The Mendelian randomization analysis revealed a causal nexus between cigarette smoking and an increased risk of aSAH, advocating for targeted smoking cessation interventions within genetically predisposed cohorts. The results regarding the relationship between alcohol consumption and aSAH were affected by insufficient statistical power. A prudent interpretation of the findings highlights the limitations of Mendelian randomization in elucidating intricate genetic epidemiological relationships. Ongoing research involving larger cohort sizes and advanced methodological approaches is essential for comprehending the genetic underpinnings of aSAH.

## Introduction

Aneurysmal subarachnoid hemorrhage (aSAH) is a severe form of stroke resulting from the rupture of an intracranial aneurysm (1). Approximately one-third of patients face mortality, while another one-third require assistance for performing daily activities. Despite advancements in aSAH care and treatment strategies, this life-threatening event still exhibits significant mortality and morbidity rates. Various studies have explored factors predicting the prognosis of aSAH (2). While genetic factors are indicated by familial dominance, lifestyle factors are considered responsible for the majority of aSAH cases (3). Smoking, a highly detrimental behavior, and exposure to cigarette smoking (CSE) are major risk factors for cerebrovascular injury, including atherosclerosis, which is a pivotal process in cerebral aneurysm (CA) formation. Smoking contributes to approximately 18% of deaths in the United States (4). It is a well-established risk factor for the development and rupture of cerebral aneurysm. Despite an improved prognosis with aggressive treatments, subarachnoid hemorrhage often leads to death or severe disability, particularly in individuals under the age of 65. Smoking is the foremost preventable cause of subarachnoid hemorrhage (SAH); this is supported by numerous studies (5, 6) that demonstrated a strong dose–response relationship. The mechanisms that link smoking to the formation and rupture of cerebral aneurysm, as well as the reversibility of this risk, remain unclear. In addition, alcohol use has been identified as a potential risk factor for aSAH (7). However, previous research findings have been inconsistent, often stemming from smaller case–control or cohort studies (8) that lack comprehensive quantitative data on alcohol consumption and abstinence. Furthermore, certain studies have been confined to specific patient subgroups based on age, gender, or occupation (3) and have focused solely on recent alcohol consumption within 24 h before the onset of the disease or exclusively on deceased patients. Observational studies have reported a positive association between smoking, alcohol consumption, and aSAH risk, acknowledging the limitations of traditional statistical methods which include potential confounders and reverse causality (9).

To overcome the inherent limitations of conventional observational studies, such as susceptibility to confounding and reverse causation, and to robustly investigate whether cigarette smoking or alcohol consumption serves as an etiological factor in aneurysmal subarachnoid hemorrhage (aSAH), we strategically employed a two-sample Mendelian randomization (TSMR) approach (10). TSMR offers a methodological advantage by leveraging genetic variants associated with exposures (cigarette smoking and alcohol consumption) as instrumental variables, thereby minimizing biases and providing insights into causal relationships. Unlike observational studies, TSMR leverages the random assortment of alleles at conception, aligning with Mendel's second law, to help overcome confounding issues prevalent in observational research. This approach, akin to randomized controlled trials, strengthened the internal validity of our investigation and enhanced the reliability of the causal inference regarding the impact of lifestyle factors on aSAH risk (11, 12). This analysis estimated the association between single nucleotide polymorphisms (SNPs) linked to cigarette smoking, alcohol consumption, and the risk of aSAH, using two independent and publicly available genome-wide association study (GWAS) datasets (13, 14).

## Methods

### Data source

Data on genetic variants linked to cigarette smoking were retrieved from the GWAS and Sequencing Consortium of Alcohol and Nicotine use (GSCAN) (Table 1) (13) (Accessed on 08 January from https://conservancy.umn.edu/handle/11299/241912). The files provided summary statistics for associations with each phenotype: alcohol consumption measured as drinks per week (*n* = 10,93,137 individuals of European descent, *n* = 1,232,091, 203 SNPs) and smoking initiation (*n* = 10,93,139 individuals of European ancestry, *n* = 941,280, 71 SNPs). Our investigation examined the outcomes of a genome-wide association study (GWAS) (Table 1) (14) on aSAH, involving individuals of European ancestry. The outcome data included individual-level genotypes from 23 distinct cohorts (accessed on 08 January from https://cd.hugeamp.org/downloads.html), which were categorized into 9 European ancestry strata based on the genotyping platform and country. Each stratum underwent a separate analysis using a logistic mixed model. The combined dataset consisted of 7,495 cases and 71,934 controls, with 4,471,083 SNPs meeting the quality control (QC) thresholds. It is essential to emphasize that all the individuals analyzed were of European ancestry.

**Table 1:** The details pf GWASs included in Mendelian randomization.

**GWAS**	**Phenotype**	**Used as**	**Participants**	**Ancestry**
Bakker et al. (14)	aSAH	Outcome	5,140 cases/71,952 controls	European
Saunders et al. (13)	Drink per week	Exposure	1,093,137	European
Saunders et al. (13)	Smoking initiation	Exposure	1,093,139	European

### Statistical analysis

In this study, genetic variants were used as instrumental variables (IVs) to estimate the causal effect of cigarette smoking on aSAH risk. These IVs were selected based on the assumptions that they (1) predicted exposures (cigarettes and alcohol), (2) were independent of confounders (e.g., BMI), and (3) did not influence the results through pathways other than weekly alcohol consumption and smoking initiation (15). Initially, SNPs associated with exposures at genome-wide significance (*p* < 5e-6) were considered (S1 and S2). After excluding the SNPs in linkage disequilibrium (LD) (r^2^ <0.001; distance <10,000 kb), independent SNPs were retained (S3 and S4). Subsequently, the remaining SNPs were cross-referenced with the aSAH GWAS database, resulting in the removal of SNPs not present in the aSAH database, which yielded a final dataset of SNPs. Finally, we obtained merged SNPs as IVs in TSMR.

In the TSMR study, we employed the inverse variance weighting (IVW) method as the primary analysis to assess the causal effect between cigarette use, alcohol consumption, and aSAH. IVW uses the Wald ratio method to calculate the exposure–outcome effect corresponding to each SNP and then conducts a weighted linear regression analysis with a forced intercept of zero. IVW achieves higher estimation accuracy and testing power when three basic assumptions are met (16).

The pleiotropy of the selected SNPs was also evaluated through the Mendelian Randomization Pleiotropy RESidual Sum and Outlier (MR-PRESSO) test. The MR-PRESSO detects outliers through a global test, computes corrected estimates for horizontal multivariate validity after outlier removal (if the *p*-value of the global test is <0.05), and assesses differences between the original and updated estimates through a distortion test (17). In addition, heterogeneity between the variance-specific estimates was tested using Cochran's Q statistic in an inverse variance-weighted model. The Cochran's Q test was performed on the remaining SNPs using the R software package to identify data heterogeneity.

As a sensitivity analysis, we performed a leave-one-out analysis and assessed the combined effect values of the remaining SNPs in conjunction with the IVW. If the combined effect was consistent with the results of the main effects analysis, it indicated that no individual SNP had a disproportionate effect on the MR analysis.

In our study, we employed the Bonferroni (18) correction for correcting multiple exposures and addressing the issue of inflated type I error rates associated with conducting multiple hypothesis tests. The Bonferroni correction is a widely accepted approach that controls the familywise error rate by adjusting the significance threshold based on the number of tests performed. Given the complexity of our investigation, which involved multiple exposures (cigarette smoking and alcohol consumption) and their potential impact on aSAH risk, it was crucial to mitigate the risk of false positives.

The Bonferroni correction set at a significance level of 0.025 (*p* = 0.05/2 = 0.025) was deliberate and aimed at ensuring a stringent criterion for statistical significance. By setting a more conservative threshold, we aimed to minimize the likelihood of observing statistically significant results purely by chance. This approach aligned with the cautious interpretation of findings and guarded against potential spurious associations. In the context of the Mendelian randomization analysis, where causal inference was a key objective, a higher level of stringency in significance testing enhanced the reliability of our results.

This methodological decision reflects our commitment to maintaining a balance between sensitivity and specificity in hypothesis testing, thereby enhancing the robustness of our conclusions regarding the causal relationships between genetic determinants of exposures and aSAH risk.

**Figure 1:**
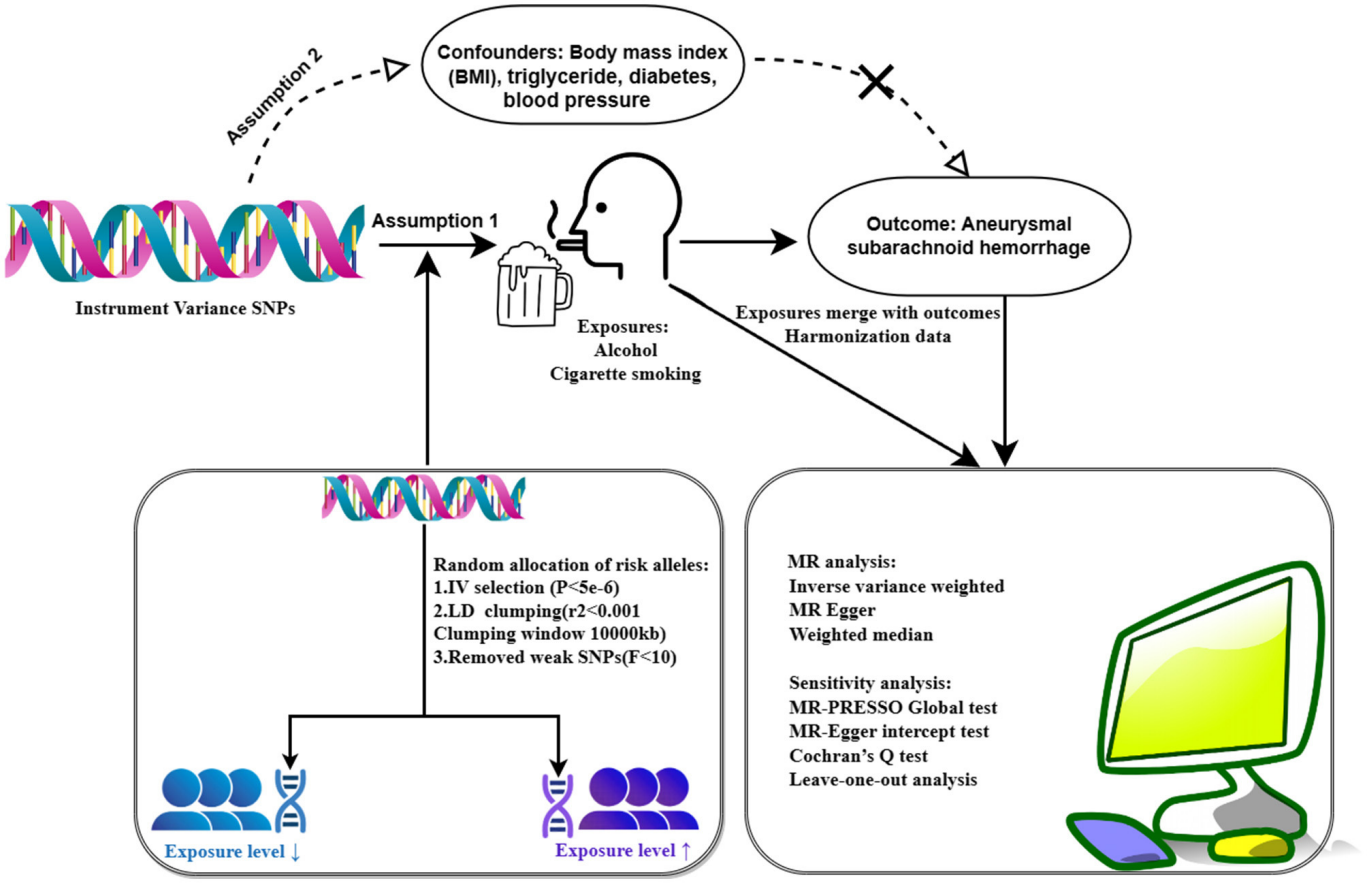
Flowchart of two sample Mendelian randomization analysis.

All analyses were conducted in R software (version 4.3.2) using the R packages, TwoSampleMR and MR-PRESSO. The results were presented as the mean effect of increased aSAH per 1-standard deviation (SD) genetic determination along with 95% confidence intervals (CIs); two-sided *p*-values <0.05 were considered statistically significant. All analytic procedures are shown in Figure 1.

## Results

### Validity of instrumental variables

To ensure the credibility of our instrumental variables (IVs) and enhance the strength of our Mendelian randomization analysis, we implemented a strict criterion for the genetic variation. Specifically, we chose a 10,000 kb limit with an r^2^ threshold of <0.001 when evaluating linkage disequilibrium (LD) between the single nucleotide polymorphisms (SNPs). This approach aimed to minimize the risk of weak instrumental variables, thereby reinforcing the validity of our instrumental variable selection.

The intentional selection of a 10,000 kb limit focused on the genetic variants in close proximity, ensuring that the chosen SNPs were more likely to be in linkage with each other. This proximity facilitated capturing potential causal relationships between the genetic variants and the exposures of interest (cigarette smoking and alcohol consumption) without introducing excessive noise from distant and less relevant genetic markers.

Simultaneously, the r^2^ threshold of <0.001 signified a low threshold for LD, indicating that the selected SNPs were relatively independent of each other. This independence was crucial for meeting the assumptions of Mendelian randomization, where instrumental variables should ideally be associated with exposures but not confounded by other factors influencing the outcome (S5–S8).

By applying these criteria, we aimed to ensure that the instrumental variables used in our analysis possessed sufficient strength and independence, addressing concerns related to weak instrument bias and enhancing the internal validity of our Mendelian randomization investigation. This process resulted in the inclusion of a total of 123 SNPs (S9: Alcohol: 30 SNPs, S10: Cigarette: 93 SNPs).

After conducting a rigorous instrumental variable screening process, we observed that all F-statistics (19) surpassed the threshold of 10, indicating robust instrumental variable strength and confirming the absence of weak instruments in our analysis. The F-statistic was a crucial metric in the instrumental variable analysis, representing the ratio of the variance explained by the instrumental variable to the unexplained variance. An F-statistic threshold exceeding 10 indicated that the chosen instrumental variables were sufficiently strong predictors of the exposure variable (cigarette smoking and alcohol consumption), ensuring that they contributed substantially to the variation in exposure. This strength was essential for fulfilling the assumptions of Mendelian randomization, where strong instruments enhance the ability to estimate unbiased causal effects. The consistent surpassing of the F-statistic threshold of 10 across all instrumental variables instilled confidence in the reliability and validity of our instrumental variable selection, reinforcing the robustness of our Mendelian randomization analysis.

### Mendelian randomization

In our Mendelian randomization investigation, we aimed to uncover the causal relationships between the genetic determinants of cigarette smoking and alcohol consumption and the risk of aneurysmal subarachnoid hemorrhage (aSAH). Overall, our study revealed compelling insights into the potential impact of these lifestyle factors on aSAH risk. Subsequently, we present the results of the inverse variance weighting (IVW) analysis for cigarettes and alcohol, shedding light on their specific associations and magnitudes.

In our two-sample Mendelian randomization (TSMR) study, we selected the inverse variance weighting (IVW) method as the primary analysis method due to its distinct advantages in estimating causal effects and enhancing statistical power. The IVW method utilizes the Wald ratio approach, calculating the exposure–outcome effect corresponding to each single nucleotide polymorphism (SNP) and conducting a weighted linear regression. This method excels in achieving higher estimation accuracy and testing power when the fundamental assumptions of Mendelian randomization are met.

The key advantages of the IVW (17) method lie in its simplicity, efficiency, and reliability. By assigning weights based on the inverse of the variance of each SNP's effect estimate, IVW maximizes the precision of the causal effect estimation. This weighting strategy, combined with a forced intercept of zero, ensures that SNPs with more precise estimates contribute more to the overall analysis, minimizing the impact of weaker instruments.

Furthermore, the IVW method is known for its statistical efficiency when applied to large-scale genetic data, making it particularly well-suited for our analysis involving a substantial number of SNPs associated with cigarette smoking and alcohol consumption. Its simplicity facilitates straightforward interpretation, which aligns with the clarity required in conveying causal relationships in complex genetic studies.

**Figure 2:**
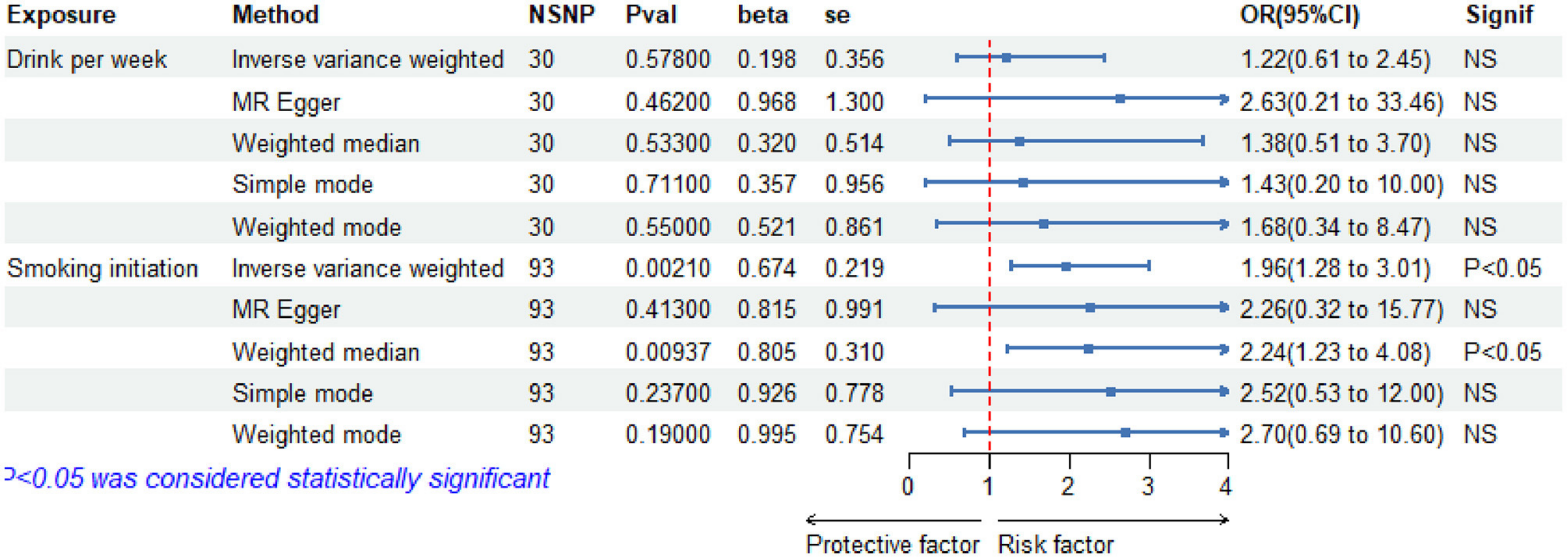
Forest plot of five Mendelian randomization estimators of the effect of alcohol and cigarette smoking on a aSAH. OR, odds ratio; CI, confidence under_val; se, standart error.

Figure 2 depicts the MR estimates for the increase in alcohol consumption and cigarette smoking and their impact on aSAH risk. In particular, the IVW results for cigarette smoking indicated a statistically significant increase in the risk of aSAH with increasing cigarette use. Employing the IVW approach, we identified a causal link between cigarette smoking and aSAH risk; a 1-standard deviation (SD) increase in the genetically determined cigarette use was causally associated with an additional 96% relative risk of aSAH (*N* = 93 SNPs; OR-IVW = 1.96; 95% CI = 1.28–3.01; and *p* = 0.0021). Conversely, alcohol consumption did not exhibit a significant association with aSAH risk (*N* = 30 SNPs; OR-IVW: 1.22; 95% CI: 0.61–2.45; and *p* = 0.578).

**Figure 3:**
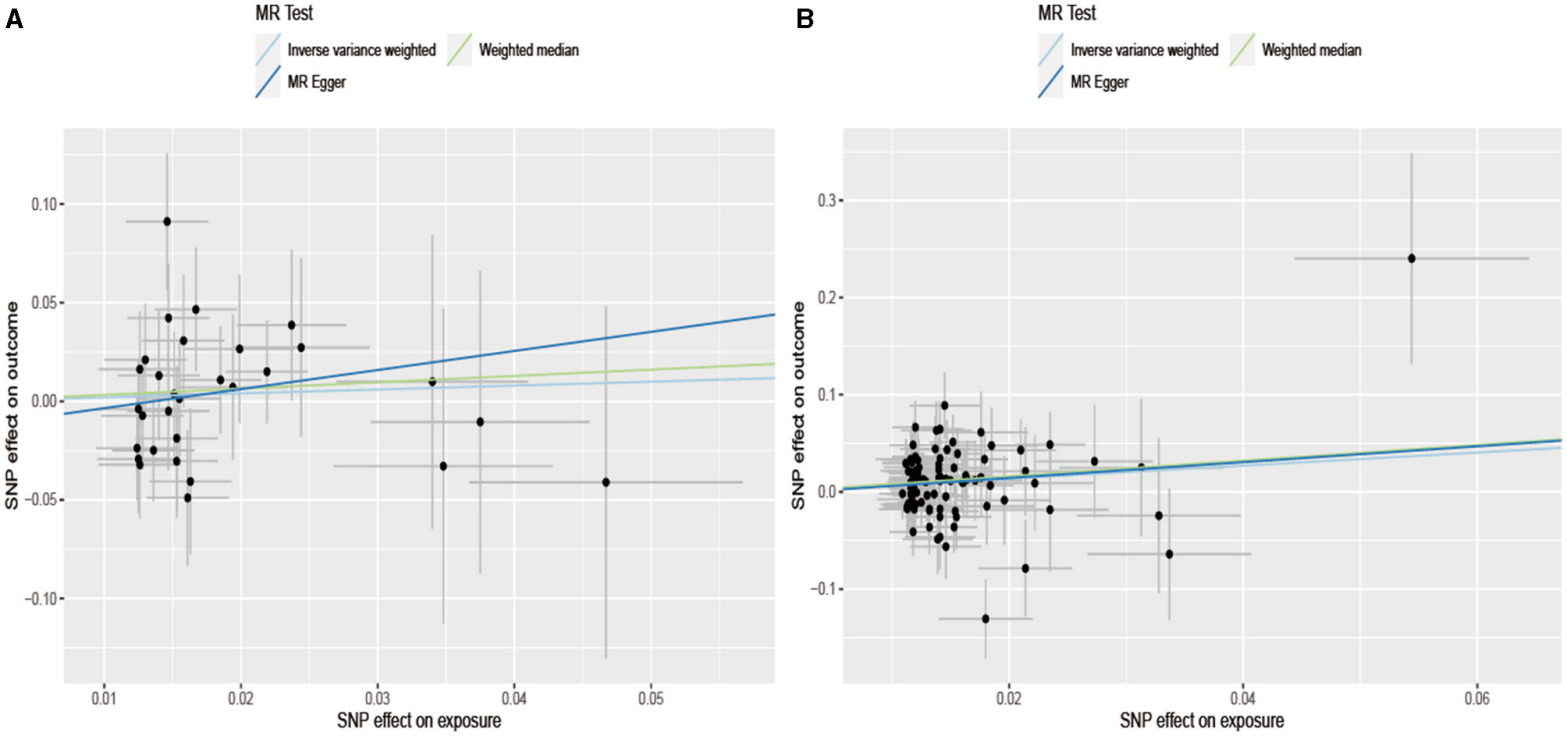
Scatterplot of SNPs associated with alcohol/cigarette and aSAH risk. The plot relates the effect sizes of the SNP-alcohol/cigarette association (x-axis, SD units) and the SNP-aSAH association [y axis, log (OR)] to 95% confidence intervals. The regression slopes of the lines correspond to causal estimates using the Mendelian randomization methods (inverse variance weighting method, MR-Egger regression, and weighted median estimator). MR, Mendelian randomization; SNP, single nucleotide polymorphism. **(A)** Drink per week; **(B)** smoking initiation.

A Mendelian randomization analysis relies on assumptions. One of these assumptions is that instrumental variables (Genetic locus) must influence the results through exposure factors. To further explore the causal relationship between these assumptions and infer the presence of horizontal pleiotropy, the MR-PRESSO Global Test (Table 2) was conducted. The results indicated no horizontal pleiotropy in the exposure factors and outcome variables (Alcohol: *p* = 0.12 and Cigarette: *p* = 0.069). Figure 3 demonstrates the absence of horizontal pleiotropy between the exposure and outcome data. It further illustrates that as cigarette consumption increased, so did the risk of coronary aSAH.

**Table 2:** The MR-PRESSO global test and heterogeneity tests of the instrumental variables.

**Exposure**	**MR-PRESSO global test**		**Cochran's test**		
	**RSSobs**	***p-*** **values**	**Q_df**	**Q_pval**	**Q**
Drink per week	45.25	0.12	31.00	0.11	40.97
Smoking initiation	26.37	0.69	29.00	0.69	24.83

**Figure 4:**
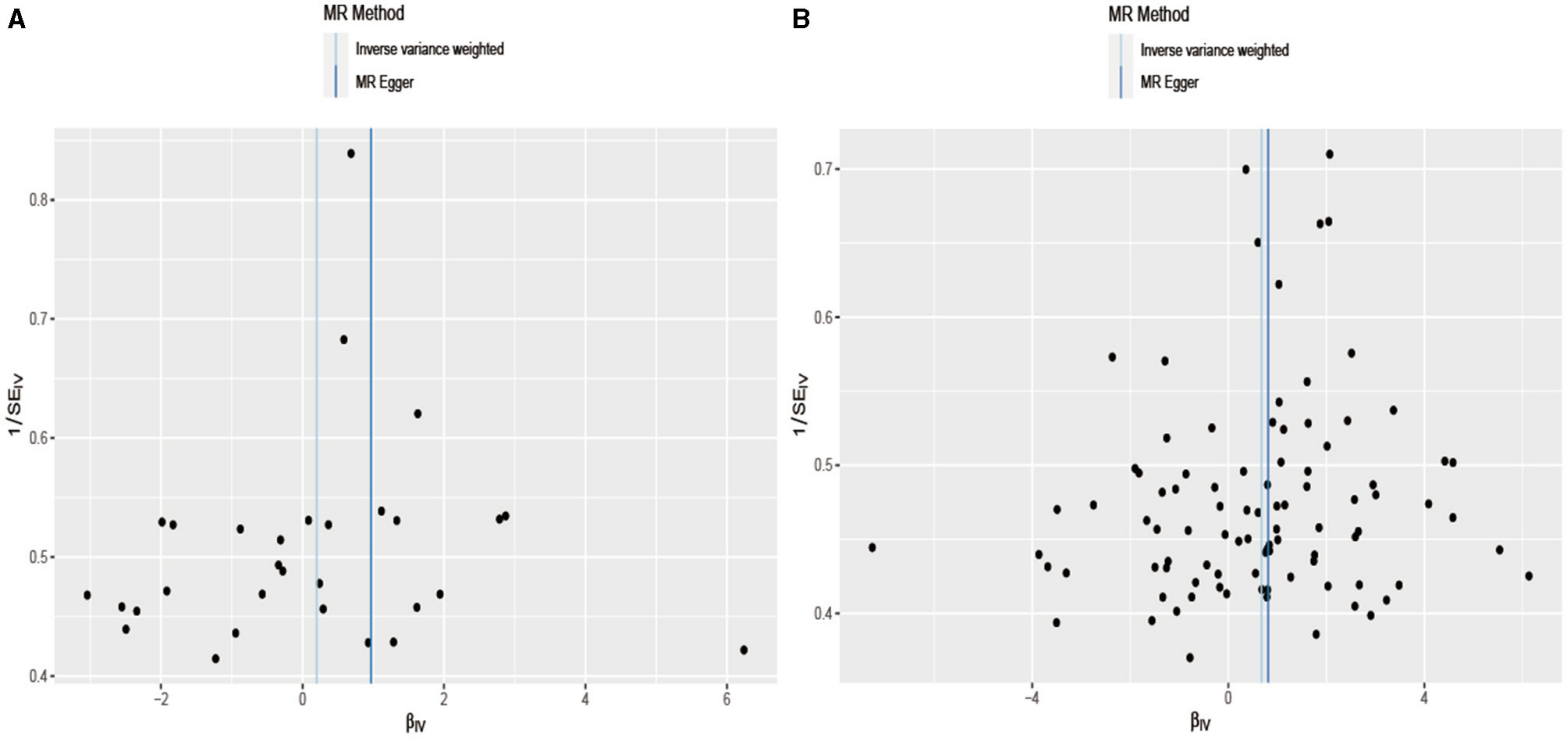
In the field of statistical analysis for research validation, a funnel plot is employed to evaluate robustness. Dispersed data points signify the effects estimated through individual SNPs serving as instrumental variables. The vertical lines represent the comprehensive estimates derived from inverse variance weighted estimated and MR-Egger regression, ensuring a through assessment of the data's integrity. **(A)** Drink per week; **(B)** smoking initiation.

Considering that the exposure and outcome data in the two-sample Mendelian randomization analysis originated from different samples, the potential population heterogeneity necessitated a heterogeneity test. Table 2 shows no significant heterogeneity between alcohol, cigarette, and aSAH (Cochran's Q test result: Q_pval of Alcohol = 0.11 and Q_pval of Cigarette = 0.69). The estimates of the random and fixed effects remained consistent without loss of precision. Figure 4 illustrates a symmetrical distribution of the effect of increased alcohol consumption and cigarette smoking on aSAH risk when a single SNP was used as the IV.

**Figure 5:**
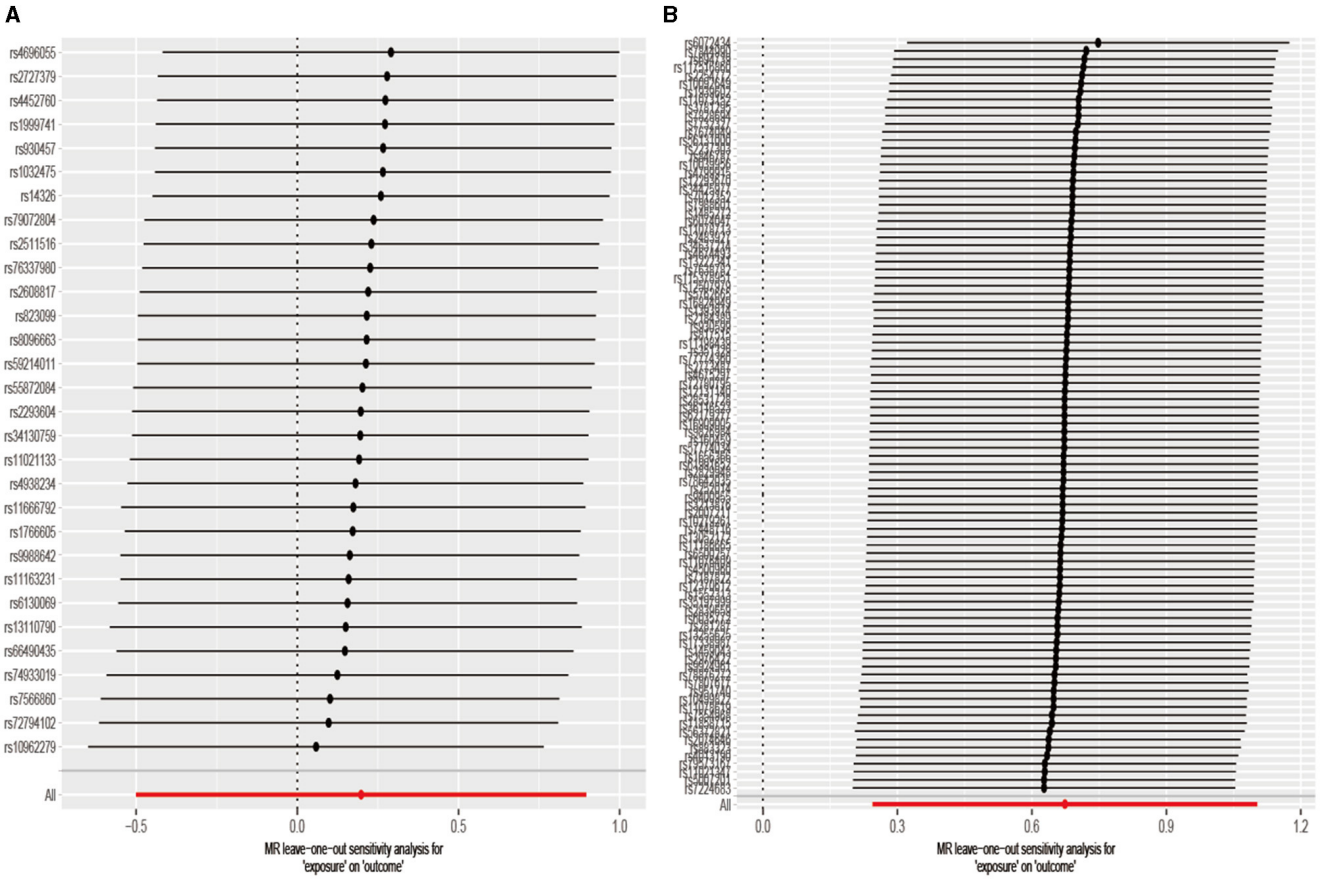
The leave-one-out method removes each SNP one by one, and then calculates the meta-effects of the remaining SNPS so as to observe whether the outcome changes significantly after the removal of a specific SNP. If the director of all beta values (>0 or <0) remained consistent, this implied that a positive causal relationship between exposure and outcomes still existed after removing any single SNP. **(A)** Drink per week; **(B)** smoking initiation.

Figure 5 shows that the leave-one-out plot precisely aligned with the right of zero (Figure 5), signifying that the outcome derived from the SNP suggested an elevated risk of developing aSAH with increased cigarette use. As for the lower red line in the chart, it signifies the IVW approach, demonstrating that cigarette use elevated the risk of aSAH.

## Discussion

### Cigarette smoking

The results of the Mendelian randomization (MR) regarding cigarette smoking revealed a significant causal relationship with an increased risk of aneurysmal subarachnoid hemorrhage (aSAH). A 1-standard deviation (SD) increase in genetically determined cigarette use was associated with an additional 96% relative risk of aSAH. These findings hold substantial implications for public health strategies and interventions. Cigarette smoking, a well-established modifiable risk factor, not only contributes to aSAH but also impacts various cardiovascular and cerebrovascular diseases. The causal link identified through the MR analysis suggests that targeted interventions to reduce cigarette smoking could potentially mitigate the risk of aSAH. Public health campaigns, especially focusing on smoking cessation in populations genetically predisposed to higher cigarette use, may significantly reduce the risk of aSAH. The observed dose–response relationship aligned with previous epidemiological evidence, emphasizing the importance of both the prevalence and intensity of smoking in tailoring effective interventions (20, 21). These results, derived from the genotypic data in the general population, are less susceptible to confounding and reverse causation compared to observational studies.

Previous research has demonstrated a dose–response relationship between smoking and SAH, but the mechanisms underlying the increased risk remain unclear. Smoking-induced transient elevations in blood pressure and chronic effects on vessel walls might contribute to aneurysm rupture. The MR results aligned with epidemiological studies, supporting a causal relationship between smoking and aSAH. However, given the complexity of cigarette smoking, the precise mechanisms are not fully understood (22). Animal studies have suggested smoking's impact on hemodynamic stress, hypertension, and vascular fragility, which are all relevant to aSAH risk. Smoking-induced atherosclerosis may affect hemodynamic stress, and a rise in blood pressure and release of enzymes may contribute to vascular fragility. The association persists even after smoking cessation, indicating potential irreversible vascular damage (23–25). Healthcare providers could incorporate genetic information on smoking susceptibility for personalized risk assessments, guiding targeted counseling and interventions. In conclusion, the MR results emphasized cigarette smoking as a causal factor in aSAH, supporting multifaceted interventions for public health.

### Alcohol consumption

Contrary to the significant association with cigarette smoking, our two-sample Mendelian randomization (TSMR) study did not reveal a significant causal link between genetically determined alcohol consumption and the risk of aneurysmal subarachnoid hemorrhage (aSAH). Several factors may have contributed to this non-significant association. The impact of alcohol consumption on aSAH risk is complex, being influenced by genetic and environmental factors. The selected genetic variants may not have fully captured these influences, which led to an incomplete representation of the causal relationship. Previous studies have shown significant associations between current and intense alcohol consumption and intracranial aneurysm rupture, but this risk diminishes after cessation (26). Methodological differences in observational studies, population characteristics, and alcohol exposure assessment may contribute to inconsistent findings (27). Mendelian randomization assumes that genetic variants solely influence the outcome through exposure, but if alternative pathways exist, it may result in a non-significant association. Alcohol flush syndrome, unrelated to alcohol consumption, has been suggested as a potential marker for aSAH risk, indicating the need for further exploration (28).

It is crucial to consider the potential threshold effects or non-linear relationships between alcohol consumption and aSAH risk. The selected genetic variants might have predominantly represented moderate alcohol consumption, while the effects at high or low levels might not have been fully captured. Acknowledging the limitations of the study, including potential population heterogeneity and unaccounted confounders, is essential. In summary, even though the TSMR study did not identify a significant causal association between genetically determined alcohol consumption and aSAH risk, the complexities in alcohol's impact, methodological considerations, and potential limitations should be considered when interpreting the results.

### Strengths and weaknesses of the study

The study's strengths include the evaluation of various lifestyle behaviors using data from a large consortium, ensuring a meticulous assessment of aSAH cases. The Mendelian randomization (MR) design mitigates reverse causality and confounding bias (29). Despite potential weak instrumental bias concerns, all exposures in the MR analyses surpassed an F-statistic threshold of 10 in a two-sample setup, minimizing this bias (30). The study employed multiple methods to ensure the robustness of the results, including weighted medians, MR-Egger, and MR-PRESSO, which addressed the issue of multiplicity in the MR analysis (31). Limiting the within-union analyses to individuals of European descent, comparing the cases to the controls at each study center, and adjusting for major components likely mitigated cohort stratification.

However, there were limitations, particularly the statistical power, which impacted the study's robustness. The study exhibited high power for smoking initiation; however, the power for the weaker associations, particularly with alcohol consumption, may have been limited. Future research with larger cohorts is crucial for detecting subtle effects and understanding genetic underpinnings comprehensively. Advancements in genetic research, including the identification of additional variants, may refine instrumental variable selection (32). Integrating multi-omics approaches and emerging genetic databases could offer novel insights into genetic architecture of aSAH. Despite the contributions made through the findings of this study, ongoing research with larger cohorts and advanced methodologies is essential.

### Methodological considerations

Mendelian randomization (MR) (33) operates on the principle of utilizing genetic variants (single nucleotide polymorphisms, SNPs) as instrumental variables (IVs) linked to exposures (cigarette smoking and alcohol consumption). This design aligns with Mendel's second law, ensuring random distribution of potential confounders and minimizing confounding bias. The temporal precedence of genetic variants precedes lifestyle behaviors, mitigating reverse causality, which is fundamental for establishing causal relationships. While MR minimizes confounding, it assumes that IVs act exclusively through exposure and are valid instruments. Deviations, such as horizontal pleiotropy, introduce bias. Sensitivity analyses, such as MR-PRESSO and the heterogeneity test address this concern.

Despite its strengths, MR has limitations. Assumptions about IVs' exclusivity and validity rely on the absence of population stratification and pleiotropy. Considering these limitations, caution in interpreting the results is essential. In conclusion, adopting the MR method strengthens causal inference, but it should be interpreted cautiously, recognizing its limitations in advancing the understanding of complex genetic epidemiology relationships.

## Data Availability

The datasets presented in this study can be found in online repositories. The names of the repository/repositories and accession number(s) can be found in the article/Supplementary material.
