# Supplementary material for: Exploring the causal links between cigarette smoking, alcohol consumption, and aneurysmal subarachnoid hemorrhage: a two-sample Mendelian randomization analysis
Source: Front Nutr. 2024 Sep 13;11:1397776. doi: 10.3389/fnut.2024.1397776 (PMC11428385; doi:10.3389/fnut.2024.1397776)
Supplement: Supplementary file 2 [file Table_2.docx]

**STROBE-MR checklist of recommended items to address in reports of Mendelian randomization studies**^1^ ^2^

| **Item No.** | **Section** | **Checklist item** | **Page No.** | **Relevant text from manuscript** |
| --- | --- | --- | --- | --- |
| 1 | **TITLE and ABSTRACT** | Indicate Mendelian randomization (MR) as the study’s design in the title and/or the abstract if that is a main purpose of the study |  | From “Exploring the Causal Links Between Cigarette Smoking, Alcohol Consumption and Aneurysmal Subarachnoid Hemorrhage：Two-Sample Mendelian Randomization” to “approaches is indispensable for comprehensive comprehension of aSAH's genetic underpinnings.” |
|  | **INTRODUCTION** |  |  |  |
| 2 | **Background** | Explain the scientific background and rationale for the reported study. What is the exposure? Is a potential causal relationship between exposure and outcome plausible? Justify why MR is a helpful method to address the study question |  | From “Aneurysmal subarachnoid hemorrhage (aSAH) is a severe form of stroke” to “including potential confounders or reverse causality.” |
| 3 | **Objectives** | State specific objectives clearly, including pre-specified causal hypotheses (if any). State that MR is a method that, under specific assumptions, intends to estimate causal effects |  | From “To overcome the inherent limitations of conventional observational studies,” to “based on two independent publicly available GWAS.” |
|  | **METHODS** |  |  |  |
| 4 | **Study design and data sources** | Present key elements of the study design early in the article. Consider including a table listing sources of data for all phases of the study. For each data source contributing to the analysis, describe the following: |  |  |
|  | a) | Setting: Describe the study design and the underlying population, if possible. Describe the setting, locations, and relevant dates, including periods of recruitment, exposure, follow-up, and data collection, when available. |  | From “Data on genetic variants linked to cigarette smoking were retrieved” to “all individuals analyzed shared European ancestry.” |
|  | b) | Participants: Give the eligibility criteria, and the sources and methods of selection of participants. Report the sample size, and whether any power or sample size calculations were carried out prior to the main analysis |  | From “Data on genetic variants linked to cigarette smoking were retrieved” to “all individuals analyzed shared European ancestry.” |
|  | c) | Describe measurement, quality control and selection of genetic variants |  | From “The study employed genetic variants as instrumental variables ” to “All analytic procedures are displayed in Figure 1.” |
|  | d) | For each exposure, outcome, and other relevant variables, describe methods of assessment and diagnostic criteria for diseases |  | From “The study employed genetic variants as instrumental variables ” to “All analytic procedures are displayed in Figure 1.” |
|  | e) | Provide details of ethics committee approval and participant informed consent, if relevant |  | From “The ethical review authority approved the studies included in the” to “an ethical review board was unnecessary. ”  ” |
| 5 | **Assumptions** | Explicitly state the three core IV assumptions for the main analysis (relevance, independence and exclusion restriction) as well assumptions for any additional or sensitivity analysis |  | From “The study employed genetic variants as instrumental variables ” to “All analytic procedures are displayed in Figure 1.” |
| 6 | **Statistical methods: main analysis** | Describe statistical methods and statistics used |  |  |
|  | a) | Describe how quantitative variables were handled in the analyses (i.e., scale, units, model) |  | From “The study employed genetic variants as instrumental variables ” to “All analytic procedures are displayed in Figure 1.” |
|  | b) | Describe how genetic variants were handled in the analyses and, if applicable, how their weights were selected |  | From “The study employed genetic variants as instrumental variables ” to “All analytic procedures are displayed in Figure 1.” |
|  | c) | Describe the MR estimator (e.g. two-stage least squares, Wald ratio) and related statistics. Detail the included covariates and, in case of two-sample MR, whether the same covariate set was used for adjustment in the two samples |  | From “The study employed genetic variants as instrumental variables ” to “All analytic procedures are displayed in Figure 1.” |
|  | d) | Explain how missing data were addressed |  | From “The study employed genetic variants as instrumental variables ” to “All analytic procedures are displayed in Figure 1.” |
|  | e) | If applicable, indicate how multiple testing was addressed |  | From “The study employed genetic variants as instrumental variables ” to “All analytic procedures are displayed in Figure 1.” |
| 7 | **Assessment of assumptions** | Describe any methods or prior knowledge used to assess the assumptions or justify their validity |  | From “The study employed genetic variants as instrumental variables ” to “All analytic procedures are displayed in Figure 1.” |
| 8 | **Sensitivity analyses and additional analyses** | Describe any sensitivity analyses or additional analyses performed (e.g. comparison of effect estimates from different approaches, independent replication, bias analytic techniques, validation of instruments, simulations) |  | From “The study employed genetic variants as instrumental variables ” to “All analytic procedures are displayed in Figure 1.” |
| 9 | **Software and pre-registration** |  |  |  |
|  | a) | Name statistical software and package(s), including version and settings used |  | From “The study employed genetic variants as instrumental variables ” to “All analytic procedures are displayed in Figure 1.” |
|  | b) | State whether the study protocol and details were pre-registered (as well as when and where) |  | N/A |
|  | **RESULTS** |  |  |  |
| 10 | **Descriptive data** |  |  |  |
|  | a) | Report the numbers of individuals at each stage of included studies and reasons for exclusion. Consider use of a flow diagram |  | From “In order to ensure the credibility of our instrumental variables (IVs) ” to “reinforcing the robustness of our Mendelian randomization analysis.” |
|  | b) | Report summary statistics for phenotypic exposure(s), outcome(s), and other relevant variables (e.g. means, SDs, proportions) |  | From “In order to ensure the credibility of our instrumental variables (IVs) ” to “reinforcing the robustness of our Mendelian randomization analysis.” |
|  | c) | If the data sources include meta-analyses of previous studies, provide the assessments of heterogeneity across these studies |  | N/A |
|  | d) | For two-sample MR:  i.  Provide justification of the similarity of the genetic variant-exposure associations between the exposure and outcome samples  ii.  Provide information on the number of individuals who overlap between the exposure and outcome studies |  | From “In order to ensure the credibility of our instrumental variables (IVs) ” to “reinforcing the robustness of our Mendelian randomization analysis.” |
| 11 | **Main results** |  |  |  |
|  | a) | Report the associations between genetic variant and exposure, and between genetic variant and outcome, preferably on an interpretable scale |  | From “In our Mendelian randomization investigation ” to “demonstrating that cigarette usage elevates the risk of aSAH.” |
|  | b) | Report MR estimates of the relationship between exposure and outcome, and the measures of uncertainty from the MR analysis, on an interpretable scale, such as odds ratio or relative risk per SD difference |  | From “In our Mendelian randomization investigation ” to “demonstrating that cigarette usage elevates the risk of aSAH.” |
|  | c) | If relevant, consider translating estimates of relative risk into absolute risk for a meaningful time period |  | From “In our Mendelian randomization investigation ” to “demonstrating that cigarette usage elevates the risk of aSAH.” |
|  | d) | Consider plots to visualize results (e.g. forest plot, scatterplot of associations between genetic variants and outcome versus between genetic variants and exposure) |  | From “In our Mendelian randomization investigation ” to “demonstrating that cigarette usage elevates the risk of aSAH.” |
| 12 | **Assessment of assumptions** |  |  |  |
|  | a) | Report the assessment of the validity of the assumptions |  | From “In our Mendelian randomization investigation ” to “demonstrating that cigarette usage elevates the risk of aSAH.” |
|  | b) | Report any additional statistics (e.g., assessments of heterogeneity across genetic variants, such as *I^2^*, Q statistic or E-value) |  | From “In our Mendelian randomization investigation ” to “demonstrating that cigarette usage elevates the risk of aSAH.” |
| 13 | **Sensitivity analyses and additional analyses** |  |  |  |
|  | a) | Report any sensitivity analyses to assess the robustness of the main results to violations of the assumptions |  | From “In our Mendelian randomization investigation ” to “demonstrating that cigarette usage elevates the risk of aSAH.” |
|  | b) | Report results from other sensitivity analyses or additional analyses |  | From “In our Mendelian randomization investigation ” to “demonstrating that cigarette usage elevates the risk of aSAH.” |
|  | c) | Report any assessment of direction of causal relationship (e.g., bidirectional MR) |  | From “In our Mendelian randomization investigation ” to “demonstrating that cigarette usage elevates the risk of aSAH.” |
|  | d) | When relevant, report and compare with estimates from non-MR analyses |  | From “In our Mendelian randomization investigation ” to “demonstrating that cigarette usage elevates the risk of aSAH.” |
|  | e) | Consider additional plots to visualize results (e.g., leave-one-out analyses) |  | From “In our Mendelian randomization investigation ” to “demonstrating that cigarette usage elevates the risk of aSAH.” |
|  | **DISCUSSION** |  |  |  |
| 14 | **Key results** | Summarize key results with reference to study objectives |  | From “The results of Mendelian randomization (MR) regarding cigarette smoking” to “limitations should be considered when interpreting results.” |
| 15 | **Limitations** | Discuss limitations of the study, taking into account the validity of the IV assumptions, other sources of potential bias, and imprecision. Discuss both direction and magnitude of any potential bias and any efforts to address them |  | From “The study's strengths include the evaluation of various lifestyle behaviors” to “advanced methodologies is essential.” |
| 16 | **Interpretation** |  |  |  |
|  | a) | Meaning: Give a cautious overall interpretation of results in the context of their limitations and in comparison with other studies |  | From “The study's strengths include the evaluation of various lifestyle behaviors” to “advanced methodologies is essential.” |
|  | b) | Mechanism: Discuss underlying biological mechanisms that could drive a potential causal relationship between the investigated exposure and the outcome, and whether the gene-environment equivalence assumption is reasonable. Use causal language carefully, clarifying that IV estimates may provide causal effects only under certain assumptions |  | From “Mendelian randomization (MR)” to “understanding of complex genetic epidemiology relationships” |
|  | c) | Clinical relevance: Discuss whether the results have clinical or public policy relevance, and to what extent they inform effect sizes of possible interventions |  | From “Mendelian randomization (MR)” to “understanding of complex genetic epidemiology relationships” |
| 17 | **Generalizability** | Discuss the generalizability of the study results (a) to other populations, (b) across other exposure periods/timings, and (c) across other levels of exposure |  | From “Mendelian randomization (MR)” to “understanding of complex genetic epidemiology relationships” |
|  | **OTHER INFORMATION** |  |  |  |
| 18 | **Funding** | Describe sources of funding and the role of funders in the present study and, if applicable, sources of funding for the databases and original study or studies on which the present study is based |  | There is no any funding in our research. |
| 19 | **Data and data sharing** | Provide the data used to perform all analyses or report where and how the data can be accessed, and reference these sources in the article. Provide the statistical code needed to reproduce the results in the article, or report whether the code is publicly accessible and if so, where |  | The data that support the findings of this study are available from the corresponding author, upon reasonable request. |
| 20 | **Conflicts of Interest** | All authors should declare all potential conflicts of interest |  | From “There are no financial relationship” to “influenced the submitted work.” |

This checklist is copyrighted by the Equator Network under the Creative Commons Attribution 3.0 Unported (CC BY 3.0) license.

1. Skrivankova VW, Richmond RC, Woolf BAR, Yarmolinsky J, Davies NM, Swanson SA, et al. Strengthening the Reporting of Observational Studies in Epidemiology using Mendelian Randomization (STROBE-MR) Statement. JAMA. 2021;under review.

2. Skrivankova VW, Richmond RC, Woolf BAR, Davies NM, Swanson SA, VanderWeele TJ, et al. Strengthening the Reporting of Observational Studies in Epidemiology using Mendelian Randomisation (STROBE-MR): Explanation and Elaboration. BMJ. 2021;375:n2233.
